# Bioinspired Cane Interface for Orientation and Mobility in Virtual Reality Using Haptic and Auditory Feedback

**DOI:** 10.3390/biomimetics11070490

**Published:** 2026-07-13

**Authors:** Jorge Clavería, Nicolas Norambuena, Damián Donoso, Jose Luis Valin, Cristobal Galleguillos

**Affiliations:** 1 Escuela de Ingeniería Mecánica, Pontificia Universidad Católica de Valparaíso, Av. Los Carrera 01567, Quilpué 2340000, Chile; jorge.claveria.t@mail.pucv.cl (J.C.); jose.valin@pucv.cl (J.L.V.); 2 Escuela de Tecnología Médica, Pontificia Universidad Católica de Valparaíso, Avenida Universidad 330, Curauma, Valparaíso 2340000, Chile; damian.donoso@pucv.cl

**Keywords:** virtual reality, virtual cane, haptic feedback, spatial audio, bioinspired design, multimodal interaction, orientation and mobility

## Abstract

This article reports the design, implementation, and formative perception-based evaluation of an early-stage virtual reality (VR) prototype that integrates a virtual cane, localized haptic feedback, 3D audio, and a three-layer bioinspired sensing framework. The prototype was implemented in Unity 2022.3 using the XR Interaction Toolkit and URP and was structured according to design science research methodology (DSRM). The bat–whisker–contact framework was used as a functional abstraction to organize distal auditory reference or warning, proximal haptic feedback, and contact confirmation; it was not evaluated against a non-bioinspired baseline. The completed evaluation consisted of an anonymous, voluntary, post-use questionnaire administered to 25 sighted participants who could select which visual-input configurations to experience. The analysis focused on reported clarity, tolerability, initial signal interpretability, and design feedback; it did not include objective navigation metrics or assess clinical efficacy, training transfer, accessibility outcomes, or orientation-and-mobility performance in blind or low-vision users. General responses suggested favorable perceived clarity and multimodal usefulness, while cane length, floor-versus-wall/obstacle differentiation, and reported discomfort identified priorities for technical refinement. In the simulated no-vision condition (n = 21), participants reported high reliance on the cane response, whereas reported initial insecurity or doubt and basic mental map ratings remained mixed. The study contributes an early-stage technological artifact and a formative basis for subsequent controlled evaluations with objective performance measures, reference conditions, and target users or orientation and mobility specialists.

## 1. Introduction

Orientation and mobility (O&M) provides one relevant application domain for the study of non-visual interaction in virtual reality. Controlled VR environments can be used to examine how auditory and haptic cues are configured, delivered, and interpreted during active exploration without treating a graphical representation of space as sufficient by itself [1,2,3]. The present work draws on this domain to develop and document an early-stage technological artifact; it does not evaluate O&M outcomes in people with visual impairments.

In non-visual interaction tasks, a cane-like interface can be understood as an active sensorimotor device through which users explore contact events, lateral boundaries, proximity information, and spatial transitions. Prior VR systems have shown that auditory and haptic cues can be integrated with virtual-cane interactions [2,3,4,5]. These precedents motivate continued engineering work on how such cues are structured within controlled experimental platforms rather than establishing that any particular configuration improves navigation or training outcomes.

From a conceptual perspective, biomimetics offers a framework for organizing staged sensing. Distal signals can provide early spatial reference or warning, proximal signals can refine the relation to nearby objects, and contact can confirm a surface or collision. Work on vibrissa-inspired tactile sensing and bat-inspired sonar has explored related principles in robotics [6,7]. In the present prototype, these biological systems are not reproduced literally; they are used as a functional abstraction for structuring auditory and haptic cues into distal reference or warning, proximal feedback, and contact confirmation.

Existing haptic–auditory VR cane systems demonstrate the value of multimodal interaction, but early-stage engineering reports still require transparent accounts of how cue hierarchies are translated into a single artifact and how formative feedback informs subsequent refinement. The gap addressed here is therefore a design and integration gap: the implementation and preliminary examination of a staged cue framework within a controlled VR/XR platform. It is not a claim that the prototype resolves an established clinical, rehabilitation, or accessibility need for blind or low-vision users.

The contribution of this work lies in implementing and documenting a single immersive artifact that combines a virtual cane, spatial 3D audio, localized haptics, visual-input restriction presets, and a bioinspired staged-sensing framework. The framework is a design hypothesis that organizes signals across distal, proximal, and contact stages. The completed study does not compare this organization with non-bioinspired or simpler multimodal designs and therefore does not establish its superiority.

Based on this design gap, the objective of this work is to develop and describe a VR/XR prototype that translates a three-layer bioinspired sensing framework into a virtual cane, spatial 3D audio, and localized haptic feedback and to conduct a formative post-use evaluation of reported perception and initial signal interpretability with sighted participants. The study is limited to artifact design, functional demonstration, and design feedback. It does not establish clinical efficacy, training transfer, accessibility outcomes, or orientation-and-mobility performance in blind or low-vision users.

**Figure 1:**
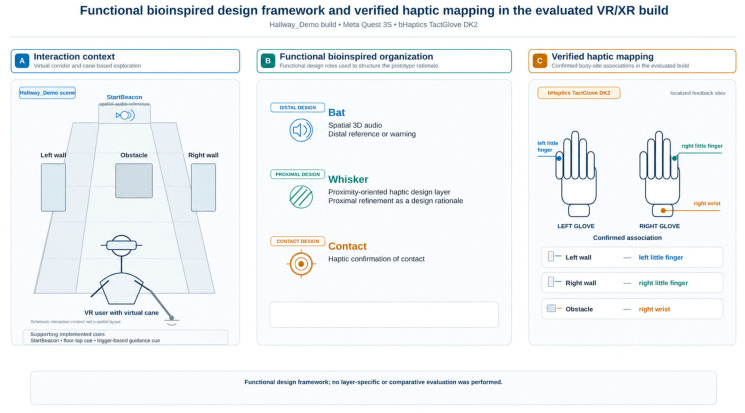
Functional bioinspired design framework and verified haptic mapping in the evaluated VR/XR build. The bat, whisker, and contact layers are presented as functional design roles used to structure the prototype rationale, whereas the mapping panel summarizes the implemented wall and obstacle haptic associations. StartBeacon, floor-tap, and trigger-based guidance cues are shown as supporting implemented cues. The figure does not represent layer-specific validation or a comparative biomimetic evaluation.

Figure 1 summarizes the functional bioinspired design framework, the verified haptic mappings, and the implemented supporting cues in the evaluated build.

## 2. Materials and Methods

This study was methodologically structured according to design science research methodology (DSRM), understood as an appropriate framework for research oriented toward the design, construction, and preliminary evaluation of technological artifacts [8,9]. In this work, DSRM was used to organize the research process and to distinguish between problem identification, objective formulation, artifact design, functional demonstration, and a preliminary evaluation of user perception.

### 2.1. Methodological Approach Based on DSRM

**Figure 2:**
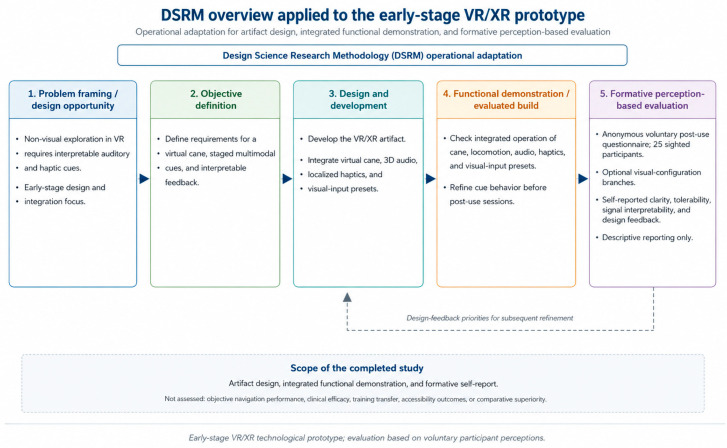
Design science research methodology (DSRM) stages adapted to the development and formative evaluation of the VR/XR prototype. The final stage consisted of an anonymous voluntary post-use questionnaire focused on reported perception and design feedback rather than objective navigation performance or clinical validation.

Following an operational adaptation of DSRM used in recent developments in virtual reality and engineering [9], and consistent with the classical formulation of the method [8], the study was organized into five stages. The first stage delimited the problem of non-visual navigation in immersive environments, the second defined the objectives of the solution, the third established the design and development of the prototype, the fourth verified its integrated operation, and the fifth enabled a preliminary evaluation through a post-use questionnaire. Figure 2 summarizes these methodological stages as adapted to the development of the prototype.

### 2.2. Problem Identification and Objective Definition

The initial phase focused on defining the research problem: non-visual navigation in virtual reality requires interpretable auditory and haptic cues, not just a graphical representation of space. This phase made it possible to formulate the design problem and establish the general criteria that the artifact had to address.

Based on this analysis, solution objectives related to safety, active exploration, multimodal feedback, and interpretability were defined. In this section, these objectives are presented as general methodological criteria; the specific technical decisions through which they were implemented are discussed later in the prototype workflow.

### 2.3. Prototype Design and Development

The design and development stage translated the methodological objectives into a functional technological artifact implemented in Unity 2022.3. The evaluated build corresponded to the Hallway_Demo scene and was run using a Meta Quest 3S headset (Meta Platforms Inc., Menlo Park, CA, USA) and a bHaptics TactGlove DK2 (bHaptics Inc., Daejeon, Republic of Korea). The prototype combined a controlled VR corridor environment, XR Interaction Toolkit and URP configuration, a virtual cane manipulated with the right hand, independent locomotion through the left controller, visual-input restriction presets, spatial 3D audio, and localized haptic feedback. The present manuscript reports the implemented interaction design and formative questionnaire results; it does not report objective navigation performance data.

The design followed the bioinspired bat–whisker–contact framework defined in the previous stages. Distal reference or warning was organized through 3D audio and StartBeacon-based spatial reference, the whisker layer was retained as a functional organization for proximity-oriented haptic design, and contact confirmation was implemented through distinct haptic mappings for wall and obstacle interactions. This section specifies the artifact-building phase of DSRM, while the detailed implementation workflow is presented in Section 3.

### 2.4. Functional Demonstration of the System

The demonstration phase was aimed at verifying that the artifact could operate coherently under controlled conditions of use. The integrated operation of the virtual cane, locomotion, visual-input restriction presets, 3D audio signaling, and localized haptic activation was checked before the formative questionnaire sessions. This phase verified integrated operation rather than outcome validation.

During this phase, iterative adjustments were made to collision behavior; cue activation; differentiation among floor, wall, and obstacle events; and the overall stability of the experience. The final build used in the questionnaire incorporated these refinements. The formative evaluation did not isolate or test the effect of individual refinements; it gathered participant feedback to identify remaining design priorities.

### 2.5. Preliminary Evaluation

The evaluation stage consisted of an anonymous, voluntary post-use questionnaire completed by 25 sighted participants. Before participation, the voluntary nature of the activity and the intended use of responses for the project were explained. No direct personal identifiers were collected. The instrument included an initial general evaluation with eight one-to-five Likert-type items addressing clarity of instructions, ease of movement, cane naturalness, discomfort or dizziness, perceived cane-length adequacy, vibration response, floor-versus-wall/obstacle differentiation, and the perceived usefulness of combined sound and vibration. It also included two open-ended questions on frustration or confusion and suggested improvements.

Participants could select which implemented visual-input configurations to experience. These configurations were not mandatory, randomized, or counterbalanced. The questionnaire used branching prompts so that condition-specific questions were answered only for configurations that the participant had actually tried. Consequently, the number of valid responses differed across unrestricted vision, the tunnel-vision preset, the central-scotoma preset, and the simulated no-vision condition.

The evaluation was conducted as a formative post-use assessment of the prototype with sighted volunteers. It was intended to gather initial information about reported signal interpretability, tolerability, and opportunities for technical refinement before any future protocol involving target users or orientation and mobility specialists. The sample was not intended to represent blind or low-vision users. Demographic characteristics, prior VR exposure, previous cane experience, motion sickness susceptibility, compensation, and other participant profile variables were not collected; this limited the interpretation of the reported perceptions and is acknowledged as a methodological limitation.

The evaluation was analyzed as a descriptive study of reported perception and formative usability feedback. Because the Likert-type responses were ordinal, results are presented through item-level response distributions, medians, and interquartile ranges, without inferential testing or between-condition comparisons. The open-ended responses were reviewed descriptively and grouped by recurring content relevant to prototype refinement; they were not subjected to a formal qualitative coding framework or intercoder-reliability assessment. The unrestricted-vision branch (n = 24) and the optional simulated no-vision branch (n = 21) provide the most informative descriptive blocks. The tunnel-vision and central-scotoma branches each had only two valid responses and are retained only to document the voluntary branching protocol; they are not interpreted as condition-level findings.

No session-level objective navigation dataset, such as travel time, collision counts, route deviation, or completion performance, was retained, exported, or analyzed in the completed formative evaluation. The present study therefore reports participant perceptions and design feedback only. Future controlled protocols should incorporate objective event logging together with subjective measures in order to examine the relation between perceived experience and navigation performance.

### 2.6. Methodological Scope

The methodological scope is limited to the construction of the artifact, its functional demonstration, and a formative perception-based evaluation with sighted volunteers. The study does not support claims of clinical validation, superiority over other mobility aids, efficacy for O&M training, training transfer, accessibility outcomes, or practical usefulness for blind or low-vision users. Its contribution is a documented early-stage technological platform and a set of design feedback observations that can guide later evaluations with stronger experimental control.

## 3. Prototype Development Workflow

**Figure 3:**
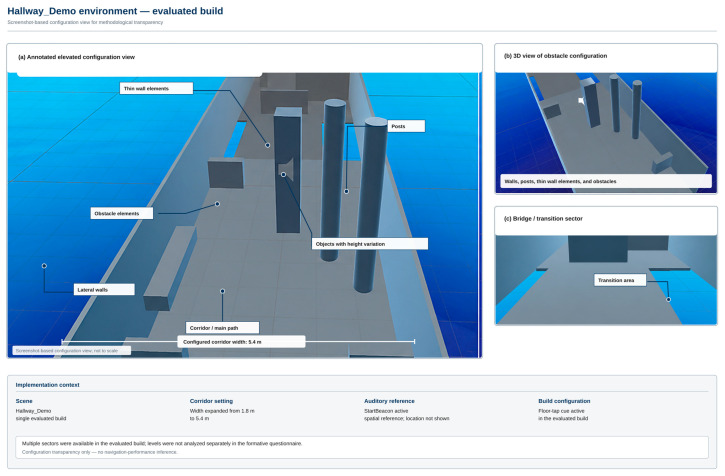
Annotated elevated configuration view of the Hallway_Demo scene used in the evaluated build. The environment included lateral walls, posts, thin walls, obstacles, modified obstacle heights, an active floor-tap cue, and the StartBeacon spatial auditory reference. Multiple sectors were available in the evaluated build; however, levels were not analyzed separately in the formative questionnaire. The figure is provided for technical transparency and does not support navigation performance inference.

As a complement to the DSRM framework, a technical workflow was defined to explain how the functional requirements of the artifact were translated into concrete implementation decisions. While DSRM organizes the research logic, the workflow focuses on the details of how the prototype was built: selection of the development engine, spatial configuration of the environment, haptic mapping, integration of 3D audio, visual-input restriction presets, and pre-evaluation refinement. For this reason, the spatial configuration of the virtual environment is presented in this section as part of the prototype development roadmap rather than as an isolated element of the overall methodology. Figure 3 shows the spatial configuration of the virtual environment as a component of the technical development roadmap.

**Figure 4:**
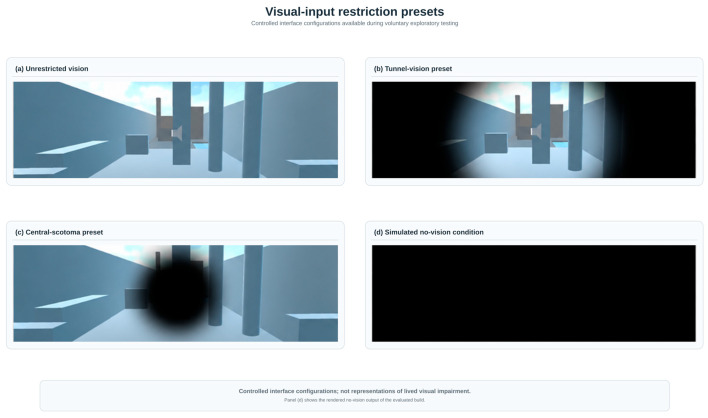
Visual-input restriction presets available during voluntary exploratory testing with sighted participants: (**a**) unrestricted vision, (**b**) tunnel-vision preset, (**c**) central-scotoma preset, and (**d**) simulated no-vision condition. These were controlled interface configurations and were not intended to reproduce the lived experience of visual impairment.

**Figure 5:**
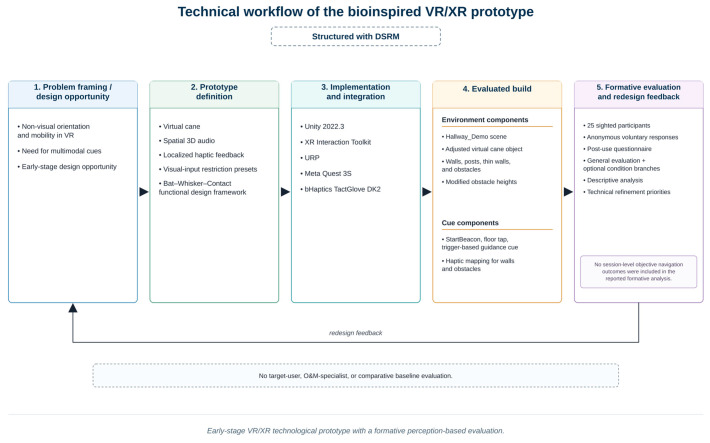
Technical development workflow of the evaluated VR/XR artifact. The workflow summarizes the translation of interaction design requirements into a controlled virtual environment, virtual-cane interaction, multimodal cues, visual-input presets, functional checks, and formative questionnaire feedback. It does not represent a performance-validation or clinical evaluation pipeline.

To make explicit the translation of biomimetics into artifact design, Table 1 distinguishes the abstract biological references, their functional design role, verified implementation features in the evaluated build, and the interpretive boundaries of the evidence. The table reports confirmed mappings and cues without assigning unverified parameters, thresholds, intensities, or layer-specific effects. Figure 4 presents the visual-input restriction presets available during voluntary exploratory testing, whereas Figure 5 summarizes the technical workflow used to translate the design requirements into the evaluated build.

### 3.1. Translation of the Problem into Requirements

The initial problem was translated into interaction requirements for representing non-visual spatial information within an immersive environment. These requirements included a virtual cane held in the right hand; independent locomotion using the left controller; differentiated handling of floor, wall, and obstacle events; directional auditory signaling; floor-tap interaction; trigger-based guidance; and visual-input restriction presets. At the conceptual level, biomimetics provided a functional abstraction of three sensing stages: distal reference or warning, proximal feedback as a design layer, and contact confirmation [6,7].

### 3.2. Iterative Implementation in Unity

Implementation was carried out in Unity 2022.3 because it supported the integration of XR Interaction Toolkit, Universal Render Pipeline (URP), scene control, visual presets, and interactive cue logic within a single development environment. The evaluated build used the Hallway_Demo scene with a Meta Quest 3S headset and a bHaptics TactGlove DK2. This choice made it possible to iteratively modify collisions, interaction logic, the world-space interface, locomotion, and cane behavior while retaining a modifiable immersive artifact. In this study, Unity is described as the implementation environment for the evaluated build rather than as evidence of technical replicability by itself.

### 3.3. Integration of Multimodal Feedback

The integration of haptics and audio was designed on the premise that non-visual interaction benefits from cues that can be distinguished by their functional role rather than from binary contact events alone. In the evaluated build, the bat layer organized spatial 3D audio and StartBeacon-based distal reference or warning. The whisker layer was retained as a functional design layer for proximity-oriented haptic organization and was not isolated empirically as an independently verified proximity mechanism. The contact layer organized haptic confirmation of wall and obstacle contact, with the left wall mapped to the left little finger, the right wall mapped to the right little finger, and obstacles mapped to the right wrist. Floor-tap and trigger-based guidance cues were active as auxiliary implemented cues. The framework did not attempt to reproduce biological sensing faithfully. Instead, it served as an engineering-oriented design abstraction for organizing progressive multimodal cues. The present evaluation did not compare individual layers or test whether the framework outperformed a non-bioinspired configuration.

### 3.4. Refinement and Preparation for Preliminary Evaluation

Before the questionnaire was administered, the prototype underwent iterative refinement of collision behavior, environmental obstacles, virtual-cane configuration, tactile differentiation between environmental events, auditory-beacon activation, floor-tap interaction, trigger-based guidance, and difficulty progression. The build evaluated by participants already incorporated these adjustments. The questionnaire was used to identify remaining issues in perceived cane length, haptic clarity, and overall tolerability; it did not test the causal effect of any single technical adjustment.

## 4. Results

### 4.1. Evaluation Instrument

**Figure 6:**
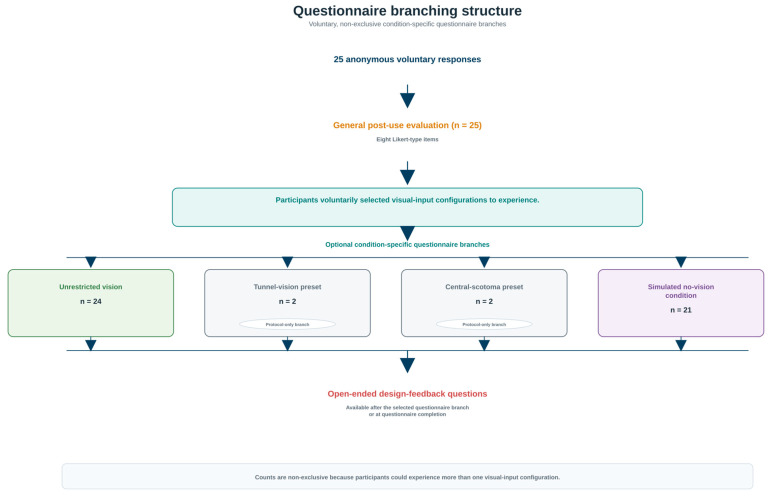
Questionnaire branching structure. All participants completed the general post-use evaluation, whereas condition-specific items were answered only for visual-input configurations voluntarily experienced by each participant. Branch counts are non-exclusive and do not represent randomized, counterbalanced, or balanced experimental groups.

The evaluation instrument was a post-use questionnaire administered in Spanish. The first section collected a general evaluation of the simulator through eight Likert-type items rated from one to five. For negatively worded items, such as discomfort or initial insecurity, higher values indicated a greater presence of the assessed experience; items were not reverse-coded for the descriptive presentation. Condition-selection prompts directed the participant through optional branches and were not analyzed as outcomes. The original Spanish wording of all items and English translations are provided in Supplementary Materials S1, together with item-level descriptive statistics for the branches retained in the main descriptive analysis. The optional branching structure and the non-exclusive availability of condition-specific responses are summarized in Figure 6.

The questionnaire structure, response availability, and analytical role of each block are summarized in Table 2.

### 4.2. Participation and Response Flow

**Figure 7:**
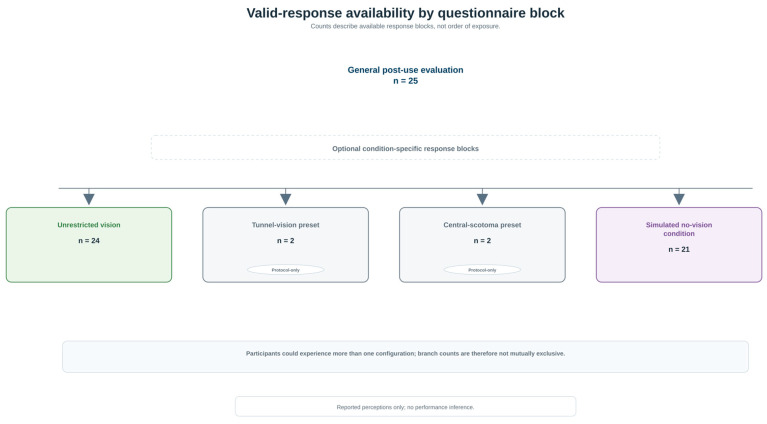
Valid-response availability across optional questionnaire branches. Counts reflect the visual-input configurations voluntarily experienced and reported by participants and are not mutually exclusive. Tunnel-vision and central-scotoma branches are retained for protocol transparency only and were not interpreted as condition-level findings.

The analyzed dataset comprised 25 anonymous responses from sighted volunteers who tested the prototype during an exploratory phase. The visual-input configurations were optional and selected by participants; therefore, completion of all conditions was not required. Across the complete branching route, 24 valid responses were available for unrestricted vision, 21 for the simulated no-vision condition, 2 for the tunnel-vision preset, and 2 for the central-scotoma preset. The order was not randomized or counterbalanced. The unequal counts resulted from participant choice and questionnaire branching, not from technical failure. The two branches with n = 2 are reported only for protocol transparency and are not used as evidence of condition-level effects. Figure 7 summarizes the valid-response availability by optional branch.

### 4.3. Main Quantitative Results

In the initial general evaluation (n = 25), the clearest descriptive pattern was high reported clarity of instructions (median = 5, IQR = 0), ease of movement (median = 5, IQR = 1), cane naturalness (median = 5, IQR = 1), and vibration response (median = 5, IQR = 0). The perceived usefulness of combined sound and vibration had a median of 4 (IQR = 1). Cane-length adequacy (median = 4, IQR = 2) and floor-versus-wall/obstacle differentiation (median = 5, IQR = 2) showed wider response dispersion, indicating heterogeneous perceptions that are relevant for refinement. The discomfort item was negatively worded: 18 participants selected the lowest category, whereas 7 selected high or very high discomfort. Because the instrument did not collect modality-specific discomfort data or objective measures, the present study cannot attribute this variation to locomotion, VR adaptation, visual-input restrictions, haptic density, audio, or another specific factor.

In the unrestricted-vision branch (n = 24), participants reported that unrestricted vision increased initial confidence in the simulator (median = 5, IQR = 1) and that they could attend to cane vibrations without visual distraction (median = 5, IQR = 1). Confidence to try another visual-input configuration also had a median of 5, although its responses were more dispersed (IQR = 1.2). These observations describe perceptions within an optional branch; they do not support comparison with other configurations.

In the optional simulated no-vision branch (n = 21), participants reported high reliance on the cane response (median = 5, IQR = 0). Ratings for initial insecurity or doubt (median = 4, IQR = 1) and basic mental map construction through touch and sound (median = 4, IQR = 1) were more mixed. These values reflect reported perceptions under a controlled absence-of-visual-input preset and should not be interpreted as navigation performance, as evidence of O&M outcomes, or as an approximation of the lived experience of blindness.

Table 3 summarizes the descriptive patterns for the general evaluation, unrestricted vision, and the optional simulated no-vision branch only. Item-level response distributions, medians, and interquartile ranges are reported in Supplementary Materials S1. Tunnel-vision and central-scotoma response counts are not included in the main result synthesis because n = 2 in each branch does not support condition-level interpretation.

**Figure 8:**
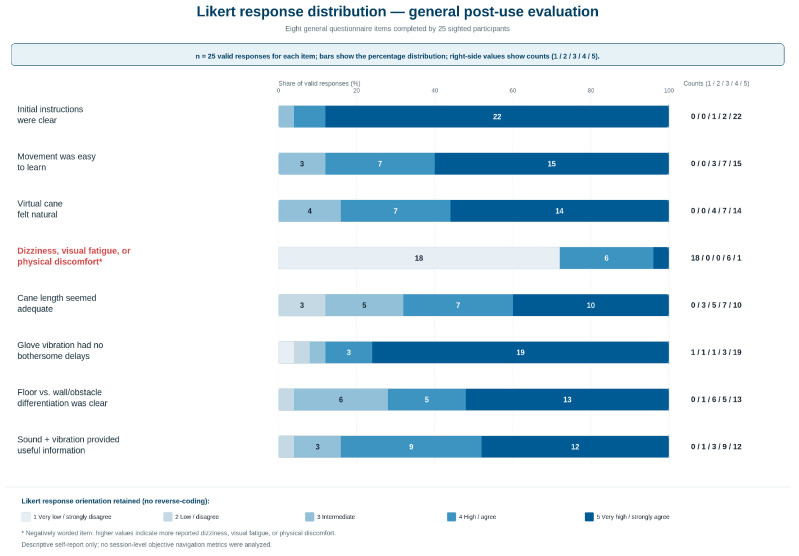
Distribution of reported Likert-scale responses in the general post-use evaluation (n = 25). The original response orientation was retained. The discomfort item is negatively worded; lower values indicate less reported discomfort, whereas higher values indicate greater reported discomfort. The figure summarizes participant perceptions and does not measure navigation performance.

**Figure 9:**
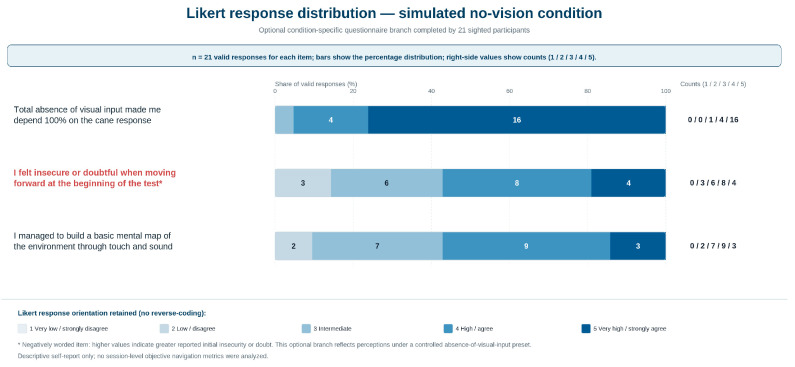
Distribution of reported Likert-scale responses in the optional simulated no-vision branch (n = 21). The original response orientation was retained. The initial-insecurity item is negatively worded; higher values indicate greater reported insecurity or doubt. This optional branch summarizes perceptions under a controlled visual-input preset and does not represent orientation-and-mobility performance or clinical outcomes.

Figure 8 and Figure 9 present the item-level Likert-response distributions for the general post-use evaluation and the optional simulated no-vision branch, respectively.

For the item on dizziness, visual fatigue, or physical discomfort, the concentration of responses in the lowest category indicates that many participants reported little discomfort. However, the simultaneous presence of high ratings shows that tolerability was not uniform across the sample. Because the questionnaire did not separate locomotion effects, VR adaptation, haptic density, audio, visual-input restriction, or other possible contributors, the source of discomfort cannot be determined from these data.

These reports identify priorities for later tolerability protocols, including progressive familiarization, planned pauses, individual calibration of the cane and multimodal feedback, and more specific recording of discomfort by modality and exposure stage. They should not be interpreted as evidence that a particular configuration caused the reported discomfort. The same applies to the optional simulated no-vision branch: the reported reliance on the cane and mixed ratings of insecurity and mental map construction identify areas for redesign rather than evidence of navigation performance or training effect.

### 4.4. Open-Ended Design Feedback Synthesis

The open-ended responses were reviewed descriptively and grouped by recurring content relevant to prototype refinement. This process was not a formal qualitative analysis, did not use a validated coding framework, and did not assess intercoder agreement. The resulting groupings should therefore be read as a transparent synthesis of design feedback rather than as a generalizable qualitative finding. Responses pointed to haptic differentiation around walls, obstacles, or perceived latency; cane length and realism; the role of sound; environmental complexity and richness; surface or drop-off differentiation; and preparation for more demanding visual-input configurations.

Table 4 presents the descriptively grouped feedback content, representative anonymized examples, and corresponding implications for prototype refinement.

### 4.5. Interpretive Synthesis of Results

Taken together, the questionnaire responses indicate that participants generally reported clear instructions and useful multimodal cues, while also identifying heterogeneous perceptions of cane length, floor-versus-wall/obstacle differentiation, haptic clarity, and discomfort. In the optional simulated no-vision branch, participants reported strong reliance on the cane response but mixed ratings of initial insecurity or doubt and basic mental map construction. These observations are most appropriately interpreted as formative design feedback for the evaluated build, not as evidence of navigation effectiveness, O&M learning, or accessibility outcomes.

## 5. Discussion

Previous work has demonstrated that virtual-cane interaction and combined haptic–auditory cues can be implemented in immersive environments [1,2,3,4,5,10,11,12,13].In addition, earlier virtual-environment systems for visually impaired users have explored orientation and mobility training and pre-journey spatial familiarization, including integrated O&M program settings and self-reliant trip-planning platforms [14,15]. The present work does not claim to exceed those systems in navigation, safety, usability, spatial learning, or mental map construction. Its narrower contribution is the implementation and documentation of an early-stage VR/XR artifact that organizes a virtual cane, spatial 3D audio, localized haptics, and visual-input restriction presets within one design science process. The formative questionnaire provides feedback about perceived interaction properties and remaining calibration issues rather than a comparison of system effectiveness.

The reported perceptions do not establish consolidated spatial exploration. In the optional simulated no-vision branch, participants reported strong reliance on the cane response while ratings of initial insecurity and basic mental map construction remained mixed. Because no objective trajectories, collision data, completion times, or learning measures were analyzed, these observations cannot be translated into claims about navigation performance. They instead indicate that cue legibility, cane configuration, haptic differentiation, and sensory transitions remain design variables requiring further refinement.

VR can serve as a controlled setting for early interaction design and subsequent testing of assistive-technology concepts [2,3,11,12]. Within that limited role, the present prototype provides a platform for examining how a staged cue hierarchy can be implemented and refined. The current evidence is restricted to a voluntary perception-based evaluation with sighted participants; it does not establish training effectiveness, transfer to real O&M contexts, or accessibility outcomes for blind or low-vision users.

In this study, the bat–whisker–contact framework served as a design scaffold rather than as a biologically faithful model. The bat layer organized distal auditory reference or warning through spatial 3D audio. The whisker layer was retained as a functional design organization for proximity-oriented haptic feedback, but it was not isolated empirically as an independently verified proximity mechanism. The contact layer organized haptic confirmation of contact. This staged arrangement informed engineering decisions about the functional role of cues, but the study did not isolate the contribution of each layer or demonstrate that the architecture improves outcomes relative to conventional multimodal feedback.

Any potential advantage of the bioinspired arrangement relative to a non-bioinspired configuration remains a design hypothesis. A future controlled evaluation should compare the complete staged architecture with clearly defined reference conditions such as contact-only, audio-only, haptic-only, or non-hierarchical multimodal feedback. Such a study would need objective navigation measures and an appropriate participant protocol before claims about the contribution of the architecture could be made.

### Limitations of the Formative Evaluation

This evaluation has several limitations. First, it relied on voluntary, anonymous self-reporting from sighted participants and did not collect demographic information, prior VR exposure, white-cane experience, motion sickness susceptibility, or other participant profile variables. Second, participants selected which visual-input configurations to experience; conditions were neither mandatory nor randomized or counterbalanced, producing unbalanced branch counts. The tunnel-vision preset and central-scotoma preset branches each had two valid responses and are therefore not interpreted as condition-level findings. Third, no session-level objective navigation dataset was retained, exported, or analyzed, including travel time, collisions, route efficiency, missed obstacles, or task completion. Fourth, the study did not include blind or low-vision users, orientation and mobility specialists, a non-bioinspired baseline, or a layer ablation comparison. Finally, the global discomfort item cannot identify whether reported discomfort was associated with locomotion, VR adaptation, visual-input restrictions, audio, haptic density, or another factor. These limitations delimit the contribution to a formative evaluation of perceived interaction properties and design feedback needs.

## 6. Conclusions

This work presented the design, implementation, and functional demonstration of an early-stage VR/XR prototype that integrates virtual-cane interaction, localized haptic feedback, spatial 3D audio, and a staged bioinspired sensing framework. The contribution is the construction and documentation of a technological artifact organized through DSRM, accompanied by a formative perception-based evaluation with sighted volunteers. It is not a validation study of O&M outcomes, clinical efficacy, accessibility, or training transfer.

The questionnaire results describe reported clarity, perceived multimodal usefulness, and design feedback priorities in the evaluated build. Participants also identified issues involving cane length, haptic differentiation of environmental events, and tolerability. In the optional simulated no-vision branch, reported reliance on the cane was high, while reported initial insecurity or doubt and basic mental map ratings remained mixed. These findings should be interpreted as requirements for prototype refinement rather than as evidence of navigation performance or practical usefulness for blind or low-vision users.

Future work should use protocols that collect objective navigation measures, apply balanced or counterbalanced conditions, and compare the staged bioinspired framework with defined reference configurations. Subsequent evaluations should also involve target users and orientation and mobility specialists under an appropriate ethical and methodological protocol. These steps are required before the contribution of the architecture, the tolerability of the system, and any potential relevance to O&M-related applications can be assessed more rigorously.

## Figures and Tables

**Table 1:** Functional bioinspired design framework and verified cue implementation in the evaluated VR/XR build.

Functional Element	Abstract Biological Reference	Design Function	Verified Implementation in the Evaluated Build	Interaction Context and Output Mapping	Evidence Presented in This Study	Main Interpretive Boundary
Bat	Bat echolocation as an abstract model of distal sensing	Organize distal spatial reference or warning before direct environmental contact	Spatial 3D audio and the StartBeacon were active in the Hallway_Demo scene used during the formative evaluation.	Reference or beacon context; spatial auditory output through the Meta Quest 3S setup	Verified build-level implementation; no cue-specific outcome analysis was performed.	Audio parameters, cue timing, and the isolated contribution of this cue to reported perceptions were not analyzed. No comparison with a non-bioinspired auditory configuration was conducted.
Whisker	Vibrissa-inspired localized sensing as an abstract model of proximal refinement	Organize proximity-oriented haptic feedback within the functional design framework	Proximity-oriented haptic feedback was retained as a functional design layer. A separate active proximity-event mapping was not verified in the active cane configuration documented for the evaluated scene.	Functional organization of localized haptic cues through the bHaptics TactGlove DK2	Framework-level design organization; no independent analysis of proximity thresholds, actuator rules, or whisker-layer effects was performed.	The whisker layer was not isolated empirically, and its contribution cannot be distinguished from other implemented haptic cues.
Contact	Direct tactile contact as an abstract model of environmental confirmation	Organize haptic confirmation of wall and obstacle contact	Left wall mapped to the left little finger; right wall mapped to the right little finger; obstacles mapped to the right wrist.	Environmental contact through the bHaptics TactGlove DK2 during wall and obstacle interactions	Verified scene-level mapping; the formative questionnaire included general reported perceptions of vibration responsiveness and ground–obstacle differentiation.	Mapping-specific performance, accuracy, and comparative benefit were not assessed. Reported perceptions do not establish navigation effectiveness.
Auxiliary implemented cues	No one-to-one biological reference assigned	Support interaction, orientation, and environmental event feedback within the evaluated build	StartBeacon, floor-tap cue, and trigger-based guidance cue were active during evaluation.	Spatial auditory reference, floor-tap interaction cue, and trigger-based guidance interaction	Verified build-level implementation; these cues were not independently analyzed as outcomes.	These features were not assigned exclusively to bat, whisker, or contact, and their individual contribution to reported perceptions was not isolated.

Note: The Hallway_Demo scene corresponded to the final build used in the formative perception-based evaluation with 25 sighted participants who provided anonymous voluntary post-use responses. This table distinguishes the bioinspired functional design framework from verified implementation features of the evaluated build. The floor-tap and trigger-based guidance cues are reported as auxiliary implemented features because no one-to-one empirical association with a single bioinspired layer was established. The whisker layer was retained as a functional design organization and was not isolated empirically as an independently verified proximity mechanism. The study did not compare biomimetic and non-biomimetic configurations, analyze objective navigation metrics, or evaluate the system with blind or low-vision users or orientation-and-mobility specialists.

**Table 2:** Structure of the ad hoc post-use questionnaire, valid-response availability, and analytical role of each block.

Questionnaire Block	Item Set/Content	Format	Valid-Response Availability	Analytical Role
General post-use evaluation	Eight items addressing instruction clarity, ease of movement, virtual-cane naturalness, dizziness/visual fatigue/physical discomfort, perceived cane-length adequacy, glove-vibration response, floor-versus-wall/obstacle differentiation, and perceived usefulness of combined sound and vibration.	Likert-type, 1–5	n = 25 for each item	Main descriptive synthesis of reported post-use perceptions and technical refinement needs.
Condition-selection and routing prompts	Five prompts directing respondents to the visual-input configuration they chose to experience.	Routing prompts; not outcome items	Not applicable	Directed the optional branches and were not analyzed as outcomes.
Unrestricted-vision branch	Initial confidence in the simulator, confidence to try another configuration, and attention to cane vibrations.	Likert-type, 1–5	n = 24 for each item	Descriptive reporting of perceptions within an optional branch.
Tunnel-vision preset branch	Perceived peripheral blockage, head movement during exploration, and perceived role of the cane in avoiding lateral contact.	Likert-type, 1–5	n = 2 for each item	Retained for protocol transparency only; not interpreted as condition-level evidence.
Central-scotoma preset branch	Reliance on peripheral vision, perceived difficulty identifying frontal obstacles, and perceived haptic compensation.	Likert-type, 1–5	n = 2 for each item	Retained for protocol transparency only; not interpreted as condition-level evidence.
Simulated no-vision condition branch	Reported reliance on the cane response, initial insecurity or doubt, and basic mental map construction through touch and sound.	Likert-type, 1–5	n = 21 for each item	Descriptive reporting of perceptions within an optional branch.
Open-ended design feedback questions	One question on frustration or confusion and one question on a suggested improvement.	Free-text response	n = 22 for frustration/confusion; n = 23 for suggested improvements	Descriptive synthesis of design feedback content for prototype refinement.

Note: The questionnaire was an ad hoc post-use instrument administered in Spanish. English translations and complete item wording are provided in Supplementary Materials S1. All participants completed the general post-use evaluation. Condition-specific branches were answered only when a participant had voluntarily selected and experienced the corresponding visual-input configuration. Branch counts are therefore non-exclusive and do not represent randomized, counterbalanced, or balanced experimental groups. The tunnel-vision and central-scotoma branches are reported only to document the questionnaire protocol.

**Table 3:** Descriptive summary of participant-reported perceptions in the main questionnaire blocks.

Evaluation Block and Item Set	Available Evidence	Descriptive Pattern	Redesign or Protocol Implication	Interpretive Limit
General post-use evaluation: clarity, interaction, and cue perception	n = 25. Median [IQR]: instruction clarity, 5 [0]; ease of movement, 5 [1]; virtual-cane naturalness, 5 [1]; glove-vibration response, 5 [0]; usefulness of combined sound and vibration, 4 [1].	Responses were concentrated toward the higher end of the scale for reported clarity, initial interaction, virtual-cane naturalness, and vibration responsiveness.	Preserve the current introductory structure while refining the cue configuration in subsequent prototype iterations.	Self-reported perceptions only. The contribution of individual audio, haptic, locomotion, or interface components was not isolated.
General post-use evaluation: refinement-related items	n = 25. Median [IQR]: cane-length adequacy, 4 [2]; floor-versus-wall/obstacle differentiation, 5 [2]; discomfort item, 1 [3]. Eighteen participants selected the lowest discomfort category, whereas seven selected high or very high discomfort.	Cane-length adequacy, event differentiation, and reported discomfort showed greater response dispersion than the core clarity items.	Prioritize adjustable cane configuration, clearer environmental-event differentiation, and future modality-specific tolerability recording.	The available data cannot attribute discomfort or differentiation difficulties to a specific technical cause and do not measure navigation performance.
Unrestricted-vision branch	n = 24. Median [IQR]: initial confidence, 5 [1]; confidence to try another configuration, 5 [1.2]; attention to cane vibrations without visual distraction, 5 [1].	Participants commonly reported confidence in the simulator and the ability to attend to cane vibrations after selecting this branch.	Consider structured familiarization before later controlled protocols.	Optional, non-comparative branch. These responses cannot be compared with other visual-input configurations.
Simulated no-vision condition branch	n = 21. Median [IQR]: reported reliance on cane response, 5 [0]; initial insecurity or doubt, 4 [1]; basic mental map construction through touch and sound, 4 [1].	Reported reliance on the cane was high, whereas initial insecurity and basic mental map ratings remained more heterogeneous.	Prioritize refinement of early-stage guidance, cue legibility, and haptic differentiation during non-visual interaction.	Perceptions under a controlled absence-of-visual-input preset only; not evidence of orientation-and-mobility performance, accessibility outcomes, training transfer, or experience of blindness.

Note: Values are reported as median [interquartile range]. Original item orientation was retained. Higher values on the discomfort and initial-insecurity items indicate greater reported discomfort or insecurity, respectively. Tunnel-vision and central-scotoma branches are not included in this primary synthesis because each had n = 2; they remain documented for protocol transparency. Complete item-level distributions are provided in Supplementary Materials File S1. No inferential testing or between-condition comparison was performed.

**Table 4:** Descriptively grouped open-ended design feedback and implications for prototype refinement.

Descriptive Feedback Grouping	Illustrative Feedback Content	Representative Anonymized Example	Implication for Prototype Refinement
No specific difficulty reported or positive appraisal	Some entries described no relevant frustration or a generally comfortable and easy-to-use experience.	“None, it is easy to use and very comfortable.”	Preserve the introductory clarity and tolerability features while examining them in later, more structured protocols.
Haptic timing, strength, and event differentiation	Entries referred to delayed, insufficient, or poorly differentiated vibration, particularly when distinguishing walls, obstacles, and the floor.	“The vibrations did not feel quite right, so I did not know whether I was against a wall or the floor.”	Review haptic timing, intensity, and differentiation rules for wall, obstacle, floor, and contact events.
Cane embodiment, geometry, and adjustability	Suggestions addressed cane length, adjustability, and a more realistic cane representation.	“The length of the cane should be adaptable to the person.”	Evaluate adjustable cane-length options and review the initial representation of the virtual cane.
Auditory contact and localization cues	Entries suggested adding or clarifying sound related to orientation, contact, or localization.	“Add sound to the cane.”	Refine the functional distinction among auditory orientation, localization, and contact cues.
Surface, path, and drop-off differentiation	Comments described uncertainty between the drop-off and the path, or they requested more continuous ground feedback.	“Maybe not being able to clearly differentiate between the drop-off and the path.”	Improve edge, transition, and ground-continuity cues in the virtual environment.
Environmental complexity and richness	Suggestions included longer paths, additional obstacles, or greater environmental richness.	“It could be an environment with more obstacles.”	Introduce progressive environmental complexity while preserving a manageable familiarization stage.
Interaction controls and visual-preset transitions	Feedback referred to visual-configuration changes and apprehension after initial wall contact in the simulated no-vision condition.	“I got scared when I hit a wall in complete darkness.”	Review visual-preset switching, preparation, and instructions before more demanding non-visual configurations.

Note: This table is a descriptive design feedback synthesis of the available Spanish-language open-ended responses. The groupings were created to organize issues relevant to prototype refinement; they do not constitute a formal qualitative coding framework, thematic analysis, prevalence estimate, or inter-rater reliability assessment. A single response could contribute to more than one descriptive grouping. Representative examples were anonymized and translated into English for reporting.

## Data Availability

The original Spanish questionnaire, English translations, and item-level descriptive statistics supporting the findings of this study are available in Supplementary Materials File S1.

## Supplementary Materials

The following supporting information can be downloaded at: https://www.mdpi.com/article/10.3390/biomimetics11070490/s1, Supplementary Materials File S1: Original Spanish post-use questionnaire with English translations and item-level descriptive statistics for the general evaluation, unrestricted-vision branch, and simulated no-vision branch.
