# Supplementary material for: Bioinspired Cane Interface for Orientation and Mobility in Virtual Reality Using Haptic and Auditory Feedback
Source: Biomimetics (Basel). 2026 Jul 13;11(7):490. doi: 10.3390/biomimetics11070490 (PMC13406556; doi:10.3390/biomimetics11070490)
Supplement: Supplementary file 1 [file biomimetics-11-00490-s001.zip › biomimetics-4389236-supplementary.pdf]

Supplementary Material S1

Post-use questionnaire and item-level descriptive statistics

Purpose. This supplementary material provides the original Spanish wording of the anonymous voluntary post-use questionnaire, English translations for reporting purposes, and descriptive statistics for the branches retained in the main manuscript. The questionnaire was administered to sighted volunteers; it was used for formative design feedback and was not a validated clinical or performance instrument.

Response scale. Likert-type responses used a 1-to-5 scale. Item orientation was preserved; negatively worded items were not reverse-coded in the descriptive reporting.

S1.1. Questionnaire structure, original Spanish wording, and English translations

General evaluation (all participants)

Item	Original Spanish wording	English translation
1	Las instrucciones iniciales para entender el simulador fueron claras.	The initial instructions for understanding the simulator were clear.
2	Moverse por el entorno virtual fue fácil de aprender.	Moving through the virtual environment was easy to learn.
3	El uso del bastón virtual se sintió natural.	Using the virtual cane felt natural.
4	Sentí mareo, fatiga visual o incomodidad física durante la prueba.	I felt dizziness, visual fatigue, or physical discomfort during the test.
5	El largo del bastón virtual me pareció adecuado para explorar el entorno.	The length of the virtual cane seemed adequate for exploring the environment.
6	La vibración en los guantes respondió sin retrasos molestos.	The vibration in the gloves responded without bothersome delays.
7	Pude diferenciar claramente el suelo de una pared u obstáculo.	I could clearly differentiate the floor from a wall or obstacle.
8	La combinación de sonidos (sonar) y vibraciones me entregó información útil.	The combination of sounds (sonar) and vibrations provided useful information.

Condition-selection prompts These prompts directed optional questionnaire branches and were not analyzed as outcome items.

Item	Original Spanish wording	English translation
9	¿Qué configuración de visión vas a evaluar primero?	Which visual configuration will you evaluate first?
13	¿Probaste otra configuración? Elige cuál evaluar ahora (o ve al final):	Did you try another configuration? Choose which one to evaluate now (or go to the end):
17	¿Probaste otra configuración? Elige cuál evaluar ahora (o ve al final): 2	Did you try another configuration? Choose which one to evaluate now (or go to the end): 2
21	¿Probaste otra configuración? Elige cuál evaluar ahora (o ve al final): 3	Did you try another configuration? Choose which one to evaluate now (or go to the end): 3
25	¿Probaste otra configuración? Elige cuál evaluar ahora (o ve al final): 4	Did you try another configuration? Choose which one to evaluate now (or go to the end): 4

Unrestricted-vision branch

Item	Original Spanish wording	English translation
10	Poder ver sin restricción me dio más confianza en el simulador.	Being able to see without restriction gave me more confidence in the simulator.
11	Me sentí con confianza para probar otro tipo de visión después de esta etapa.	I felt confident to try another type of vision after this stage.
12	Pude concentrarme en las vibraciones del bastón sin que la visión me distrajera.	I could focus on the cane vibrations without vision distracting me.

Tunnel-vision branch Only two valid responses were available; no condition-level analysis was performed.

Item	Original Spanish wording	English translation
14	La viñeta negra bloqueó efectivamente mi visión periférica y no pude evadirla.	The black vignette effectively blocked my peripheral vision and I could not bypass it.
15	Me sentí obligado a mover físicamente la cabeza constantemente para explorar.	I felt compelled to physically move my head constantly to explore.
16	El uso del bastón fue indispensable para evitar chocar lateralmente.	Using the cane was indispensable for avoiding lateral collisions.

Central-scotoma branch Only two valid responses were available; no condition-level analysis was performed.

Item	Original Spanish wording	English translation
18	El punto ciego central me obligó a depender mucho más de mi visión periférica.	The central blind spot forced me to depend much more on my peripheral vision.
19	Identificar obstáculos justo frente a mí fue un desafío realista.	Identifying obstacles directly in front of me was a realistic challenge.
20	Sentí que las alertas hápticas del bastón compensaron la falta de visión central.	I felt that the cane's haptic alerts compensated for the lack of central vision.

Simulated no-vision branch

Item	Original Spanish wording	English translation
22	La falta total de visión me hizo depender 100% de la respuesta del bastón.	The total absence of vision made me depend 100% on the cane response.
23	Sentí inseguridad o duda al avanzar al principio de la prueba.	I felt insecure or doubtful when moving forward at the beginning of the test.
24	Logré construir un mapa mental básico del entorno guiándome solo por el tacto y el sonido.	I managed to build a basic mental map of the environment guided only by touch and sound.

Open-ended questions

Item	Original Spanish wording	English translation
26	¿Hubo algún momento en que el simulador te causó frustración o confusión? ¿Cuál?	Was there any moment when the simulator caused you frustration or confusion? Which one?
27	Si pudieras mejorar una sola cosa de la experiencia, ¿qué sería?	If you could improve one thing about the experience, what would it be?

S1.2. Item-level descriptive statistics

Analytical note. The statistics below are descriptive only. Medians and interquartile ranges are reported for the general evaluation, unrestricted-vision branch, and simulated no-vision branch. Tunnel vision and central scotoma are excluded from condition-level statistics because each branch had n = 2.

General evaluation (n = 25)

Item (English shorthand)	n	Median	Q1-Q3	IQR	Counts (1/2/3/4/5)
The initial instructions for understanding the simulator were clear.	25	5	5-5	0	0/0/1/2/22
Moving through the virtual environment was easy to learn.	25	5	4-5	1	0/0/3/7/15
Using the virtual cane felt natural.	25	5	4-5	1	0/0/4/7/14
I felt dizziness, visual fatigue, or physical discomfort during the test.	25	1	1-4	3	18/0/0/6/1
The length of the virtual cane seemed adequate for exploring the environment.	25	4	3-5	2	0/3/5/7/10
The vibration in the gloves responded without bothersome delays.	25	5	5-5	0	1/1/1/3/19
I could clearly differentiate the floor from a wall or obstacle.	25	5	3-5	2	0/1/6/5/13
The combination of sounds (sonar) and vibrations provided useful information.	25	4	4-5	1	0/1/3/9/12

Unrestricted-vision branch (n = 24)

Item (English shorthand)	n	Median	Q1-Q3	IQR	Counts (1/2/3/4/5)
Being able to see without restriction gave me more confidence in the simulator.	24	5	4-5	1	0/0/1/7/16
I felt confident to try another type of vision after this stage.	24	5	3.8-5	1.2	1/0/5/3/15
I could focus on the cane vibrations without vision distracting me.	24	5	4-5	1	0/3/1/6/14

Simulated no-vision branch (n = 21)

Item (English shorthand)	n	Median	Q1-Q3	IQR	Counts (1/2/3/4/5)
The total absence of vision made me depend 100% on the cane response.	21	5	5-5	0	0/0/1/4/16
I felt insecure or doubtful when moving forward at the beginning of the test.	21	4	3-4	1	0/3/6/8/4
I managed to build a basic mental map of the environment guided only by touch and sound.	21	4	3-4	1	0/2/7/9/3

Source note. Compiled from the de-identified form-response export provided by the authors. No direct identifiers are reproduced in this supplementary material.
